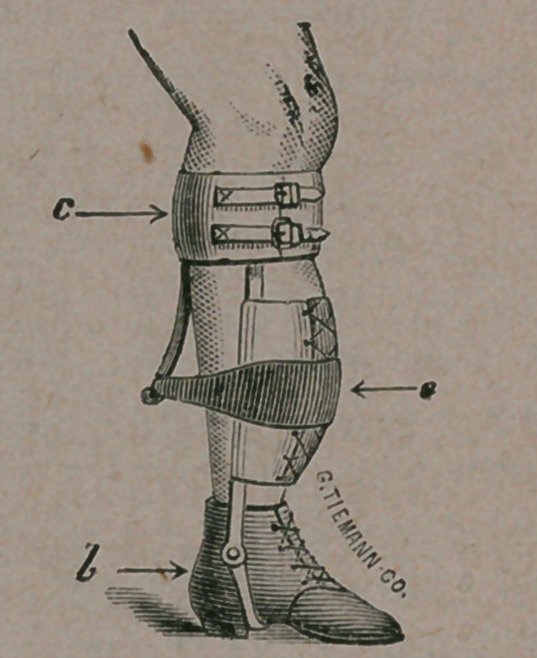# Children’s Legs

**Published:** 1878-07

**Authors:** 


### CHILDREN'S LEGS.

A lady applied to us the other day, with a bright little boy of four years of age, whose right leg bent forward in such a manner as not only to make him limp, but presented an unsightly deformity. She informed us that the leg commenced bending as soon as he began to walk, and gradually increased up to the present time.

"Why have you not done something for the child before now?" we ventured to remark.

"Because we did not know that anything could be done, and then our neighbors all told us he would outgrow it," she replied.

Only another instance of the meddlesome interference of people who assume to know everything upon subjects upon which they are entirely ignorant. We have resolved that if parents are not familiar with the manner of remedying such deformities, it shall not be the fault of the BISTOURY. In the case referred to, we applied an apparatus consisting of two upright steel bars, rivited to the sole of the shoe, and terminating in a band that buckled about the leg, just below the knee.

The bars were jointed at b, to permit free motion in the ankle joint. At *c*, a spur of steel was rivited to the steel band, under the cushion, passing down as far as the most prominent point in the bend of the leg. To this an elastic band was fastened, that passed over the curve at *a*, and constantly pulled in the opposite direction. By such a simple contrivance do we expect to render the leg perfectly straight, within two or three months, and by such or similar means can all such deformities be remedied in children, in the hands of skillful surgeons, anywhere.

Parents should never permit a deformity of any sort, to go unexamined by a surgeon, hoping that it will be outgrown; neither should they listen to meddlesome neighbors, who always volunteer advice, in cases of this character. Apply to your surgeon early, and so save yourself much anxiety, and perhaps your child from a deformity, that may cripple him for a lifetime.

## Figures and Tables

**Figure f1:**